# Azygos lobe-a rare anatomical variant

**DOI:** 10.11604/pamj.2025.50.81.44865

**Published:** 2025-03-20

**Authors:** Aishwarya Kishor Kedar, Vivek Alone

**Affiliations:** 1 Department of Respiratory Medicine, Datta Meghe Institute of Higher Education and Research, Wardha, Maharashtra, India

**Keywords:** Azygos fissure, azygos lobe, azygos vein, triagonum parietale

## Image in medicine

A sixty-three-year-old woman complained of a growth over her right buccal mucosa that was non-healing and painful. The ulcerative growth steadily became larger, and the patient also lost a substantial amount of weight and her appetite. Other than that, she had no complaints. She was vitally stable, had no concomitant illnesses, and had a history of tobacco use dating back five years. A diagnosis of moderately differentiated squamous cell carcinoma of the right buccal mucosa was obtained based on an incisional biopsy collected from the ulcerative growth. Chest radiography was carried out as a part of routine investigation which revealed a fine line in the right upper zone suggestive of an azygos fissure which separated the azygos lobe on its medial side from the rest of the right upper lobe and the lowermost part appeared teardrop-shaped which represented the azygos vein. Contrast-enhanced computed tomography of the lungs was done in order to rule out distant metastasis and confirm the presence of an azygos lobe. It revealed an azygos fissure that separated the azygos lobe from the right upper lobe. The uppermost part of the fissure had “triagonum parietale”. “Triagonum parietale” is triangular in shape and present at the uppermost part of the fissure and it contains a small amount of areolar tissue between the parietal layers of pleura. The lowermost portion of the azygos fissure had the azygos vein. There was no evidence of metastasis. Patient was further planned for surgical resection of the growth of the right buccal mucosa. An azygos lobe is an uncommon congenital variation that mainly affects the right lung. It is observed in 0.3% of the population. Although the azygos lobe is an accidental discovery and normally has little clinical significance, clinicians, particularly thoracic surgeons, should be aware of this aberration before performing the surgeries.

**Figure 1:**
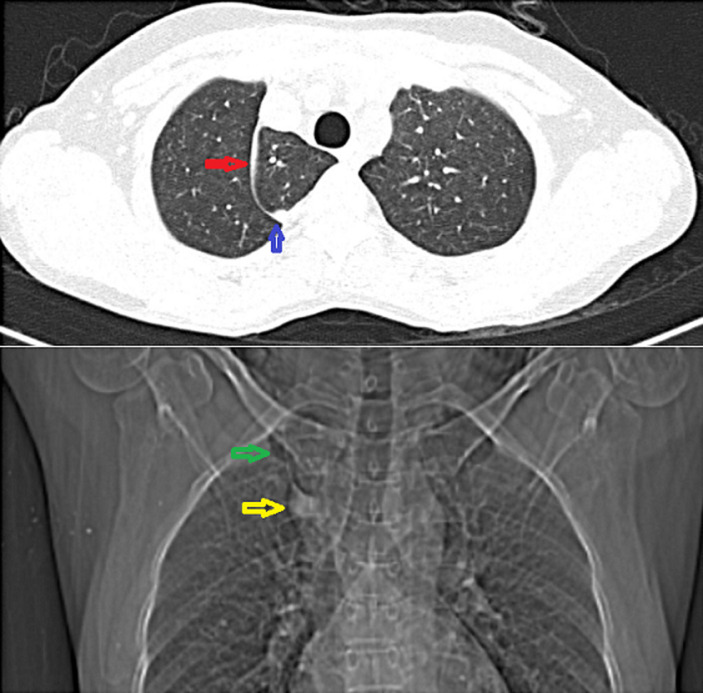
red arrow: an axial section of contrast-enhanced computed tomography of lungs showing azygos lobe separated from right upper lobe by azygos fissure; blue arrow: lower most part shows azygos vein; green arrow: zoomed in image of chest radiography showing azygos lobe along with azygos fissure; yellow arrow: azygos vein on right side

